# Shared Sulfur Mobilization Routes for tRNA Thiolation and Molybdenum Cofactor Biosynthesis in Prokaryotes and Eukaryotes

**DOI:** 10.3390/biom7010005

**Published:** 2017-01-14

**Authors:** Silke Leimkühler, Martin Bühning, Lena Beilschmidt

**Affiliations:** Department of Molecular Enzymology, Institute of Biochemistry and Biology, University of Potsdam, 14476 Potsdam, Germany; buehning@uni-potsdam.de (M.B.); beilschm@uni-potsdam.de (L.B.)

**Keywords:** tRNA, molybdenum cofactor, persulfide, thiocarboxylate, thionucleosides, sulfurtransferase, l-cysteine desulfurase

## Abstract

Modifications of transfer RNA (tRNA) have been shown to play critical roles in the biogenesis, metabolism, structural stability and function of RNA molecules, and the specific modifications of nucleobases with sulfur atoms in tRNA are present in pro- and eukaryotes. Here, especially the thiomodifications xm^5^s^2^U at the wobble position 34 in tRNAs for Lys, Gln and Glu, were suggested to have an important role during the translation process by ensuring accurate deciphering of the genetic code and by stabilization of the tRNA structure. The trafficking and delivery of sulfur nucleosides is a complex process carried out by sulfur relay systems involving numerous proteins, which not only deliver sulfur to the specific tRNAs but also to other sulfur-containing molecules including iron–sulfur clusters, thiamin, biotin, lipoic acid and molybdopterin (MPT). Among the biosynthesis of these sulfur-containing molecules, the biosynthesis of the molybdenum cofactor (Moco) and the synthesis of thio-modified tRNAs in particular show a surprising link by sharing protein components for sulfur mobilization in pro- and eukaryotes.

## 1. Introduction

Sulfur is an essential element to all living organisms and the presence of sulfur in cofactors was discovered more than a century ago [1]. The trafficking and delivery of sulfur to cofactors and nucleosides is a highly regulated process, which occurs by complex sulfur relay systems involving numerous proteins in reactions that still remain partially unknown [2,3]. In the last few years, several studies on sulfur transfer pathways in the cell concluded that the major enzymes involved in the mobilization of sulfur are l-cysteine desulfurases and/or rhodanese homology domain proteins [3,4]. While l-cysteine desulfurases are well characterized and use l-cysteine as their sulfur source [3,4,5], the role of rhodaneses in the cell often remains elusive. Rhodaneses are characterized by a 6-amino acid active-site loop with a conserved cysteine, and in vitro catalyze the transfer of a sulfane sulfur atom from thiosulfate to cyanide [3,6]. Both enzymes catalyze the formation of a persulfide group (R–S–SH) on specific conserved cysteine residues, which serves as a sulfur donor for the biosynthesis of numerous und diverse sulfur-containing biomolecules. 

In particular, l-cysteine desulfurases serve as primary sulfur-providing protein for biomolecules including FeS clusters, thiamin, biotin, lipoic acid, molybdenum cofactor (Moco), and thiolated nucleosides in tRNA, which all can be synthesized de novo in bacteria. However, in eukaryotes, de novo biosynthesis can only be accomplished for FeS clusters, Moco and transfer RNA (tRNA) thiolation [3,7,8,9]. The terminal sulfur of the persulfide group thereby can form a disulfide bond (R–S–S–R) with a target molecule as intermediate [1,6]. This provides a route for cellular sulfur transfer from a donor protein to an acceptor protein without increasing the soluble sulfur concentrations to toxic levels. Many of the sulfur-relay proteins are highly conserved across species, the most important example that initiates the sulfur transfer step being the housekeeping l-cysteine desulfurase iron sulfur cluster synthesis protein (IscS) from *Escherichia coli* that shares 60% amino acid sequence identity with its human nitrogen fixation 1 homolog (NFS1) [3]. l-cysteine desulfurases are pyridoxal phosphate-containing homodimers, which convert l-cysteine to l-alanine and sulfane sulfur via the formation of an enzyme-bound persulfide intermediate [5]. Further, the persulfide sulfur can be transferred to various acceptor proteins, which are, in general, specific for each sulfur-containing biomolecule. The fact that the biosynthesis of Moco and the synthesis of thio-modified tRNAs share protein components essential for sulfur mobilization in pro- and eukaryotes for both pathways, reveals a surprising link regarding the biosynthesis of these sulfur-containing molecules.

Thiomodifications of tRNAs were shown to be important for proper function of pro- and eukaryotic organisms [10,11]. In particular, four different thionucleosides have been identified at different positions in several prokaryotic tRNAs to date: 4-thiouridine at position 8 (s^4^U8), 2-thiocytidine at position 32 (s^2^C32), 5-methylaminomethyl-2-thiouridine at position 34 (mnm^5^s^2^U34) and 2-methylthio-N6 isopentenyladenosine at position 37 (ms^2^i^6^A37) [10] (Table 1). Among those modifications, especially the xm^5^s^2^U34 modifications are present in all three domains of life. In eukaryotes, the wobble bases of tRNAs for Glu, Gln and Lys are modified and sulfurated to form 5-methyl-2-thiouridine derivatives (xm^5^s^2^U), the most important being the 5-methoxycarbonylmethyl-2-thiouridine (mcm^5^s^2^U) modification in eukaryotic cytoplasmic tRNAs [12]. The mcm^5^s^2^U34 modification in the cytosol of eukaryotes is highly conserved and was identified to involve the same protein components in humans, yeast and plants [11]. An additional thio-modification forming 5-taurinomethyl-2-thiouridine (τm^5^s^2^U34) has been identified, the synthesis of which requires different protein components. However, this modification seem to be restricted to mammalian mitochondria only, since in other eukaryotes, including yeast, a cmnm^5^s^2^U34 tRNA modification is present in mitochondria [13] (Table 1). 

**Figure 1:**
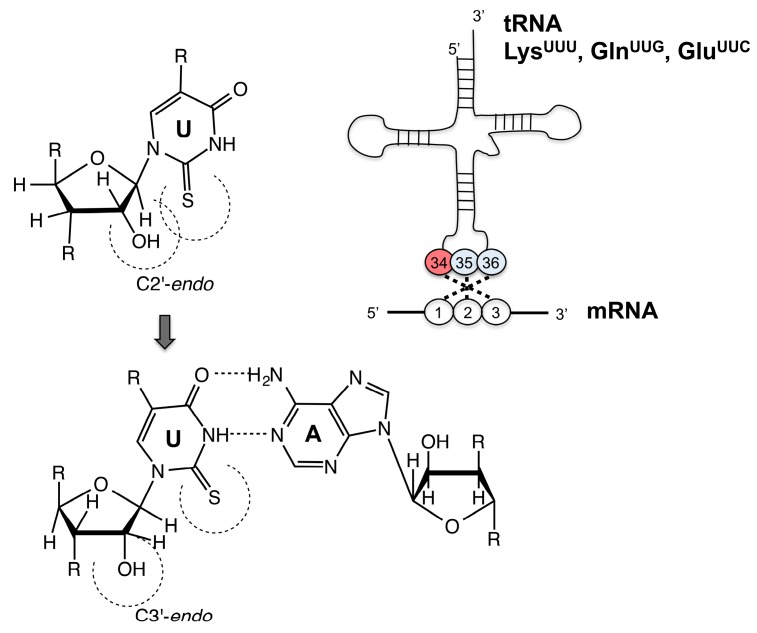
Structure and base pairing of tRNA. Conformation of the xm^5^s^2^U: C3′-*endo* form is preferred because of the steric hindrance of the 2-thio and 2′-OH groups. mRNA: messenger RNA.

The role of the thio-modification at the wobble position U34 of nucleotides present in tRNAs for lysine, glutamine or glutamate was suggested to be responsible for enhanced translation efficiency by enhancing aminoacylation kinetics, assisting proper codon–anticodon pairing and preventing frameshifting during translation [11,14]. These thio-modifications result in a conformation in which xm^5^s^2^U is trapped in the C3′-*endo* form of the ribose, since the large van der Waals radius of the 2-thio group causes a steric clash with its 2′-OH group [12,15] (Figure 1). This conformational rigidity causes preferential pairing of the xm^5^s^2^U modified bases with purines, and prevents misreading of codons ending in pyrimidines [15,16,17]. Furthermore, the 2-thio group ensures a higher stability of tRNA binding to the ribosomal A site, and prevents frameshifting during translation [18]. Studies in yeast showed that mutants lacking the 2-thio modifications have pleiotropic phenotypes, including defects in invasive growth, hypersensitivity to high temperatures, and oxidative stress. In addition, these mutants are prone to protein misfolding and aggregation, thereby being unable to maintain normal metabolic cycles [19,20,21]. In humans, the impaired 2-thio modification of the mitochondrial tRNAs has been associated with acute infantile liver failure, myoclonic epilepsy with ragged-red fibers, hypertrophic cardiomyopathy and lactic acidosis [22,23,24].

In eukaryotes, and in particular in mammals, the pathway for thio-modification of τm^5^s^2^U34 in mitochondrial tRNAs rather resembles the bacterial (c)mnm^5^s^2^U34 tRNA modification pathway. In the eukaryotic modification pathway, the mitochondrial tRNA-specific 2-thiouridylase (MTU1) protein shares amino acid sequence homologies to the 5-methylaminomethyl-2-thiouridine synthase A (MnmA) protein of bacteria [10]. In contrast, the mcm^5^s^2^U34 modification in the cytosol of eukaryotes involves the proteins molybdenum cofactor synthesis protein 3 (MOCS3), ubiquitin-related modifier 1 (URM1) and the FeS protein cytoplasmic tRNA 2-thiolation protein 1 (CTU1) as well as CTU2 in humans [25]. While this tRNA thiolation pathway is different from the mnm^5^s^2^U34 thiolation pathway in bacteria, similar proteins are present in thermophilic bacteria and archaea. The mcm^5^s^2^U34 modification pathway, in particular, is directly linked to the biosynthesis of Moco in humans, sharing the MOCS3 protein as an essential part in both pathways. 

Moco is an important molecule for life on earth, since molybdenum-containing enzymes catalyze crucial redox reactions in global metabolic cycles [26]. In Moco, the molybdenum atom is coordinated to a dithiolene group present in the pterin-based 6-alkyl side chain of molybdopterin (MPT) [27]. Many enzymes coordinating Moco have been identified. In total, bacteria contain the largest variety of more than 60 different molybdoenzymes being involved in specific, however, usually non-essential redox-reactions [28]. 

In contrast, in humans, only four different molybdoenzymes have been identified, namely sulfite oxidase, being essential to humans; in addition to xanthine dehydrogenase, aldehyde oxidase and the mitochondrial amidoxime reducing compound (mARC) [29]. A defect in Moco biosynthesis is lethal due to the loss of sulfite oxidase activity. A therapy for Moco deficiency of patients has been developed [30], however, to date, no effective therapy exists for the treatment of isolated sulfite oxidase deficiency.

This review will describe the shared sulfur mobilization routes for the biosynthesis of thiolated tRNAs and Moco with a particular focus on the pathways characterized in *E. coli* and humans.

## 2. Overview of Moco Biosynthesis in Bacteria and Mammals

**Figure 2:**
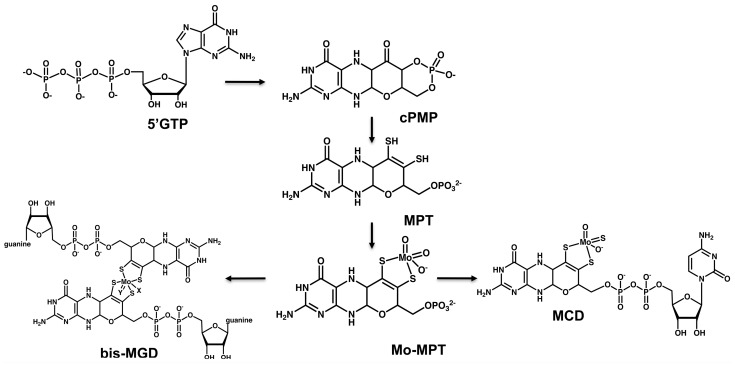
Scheme of the biosynthesis of Moco. Shown are the structures of the stable intermediates of the biosynthesis of Moco. The central part shows the three conserved steps of Moco biosynthesis present in all organisms, the formation of cPMP, MPT and Mo-MPT from 5′GTP. In bacteria, Mo-MPT can be further modified by the addition of GMP or CMP, forming the *bis*-MPT guanine dinucleotide cofactor (*bis*-MGD) or the MPT cytosine dinucleotide cofactor (MCD), respectively.

Moco is a tricyclic pyranopterin containing a unique dithiolene group to which the molybdenum atom is coordinated [31]. In general, the biosynthesis of Moco can be divided into three steps in eukaryotes, and four steps in bacteria and archaea (Figure 2): (i) the starting point is the formation of the cyclic pyranopterin monophosphate (cPMP) from 5′GTP; (ii) in the second step, two sulfur molecules are inserted into cPMP leading to the formation of molybdopterin (MPT); (iii) in the third step, the molybdenum atom is inserted into molybdopterin to form Moco; and (iv) additional modification of Moco occurs in bacteria and archaea with the attachment of a nucleotide (cytidine monophosphate (CMP) or guanosine monophosphate (GMP)) to the phosphate group of MPT, forming the dinucleotide variants of Moco. From these dinucleotide variants, the *bis*-molybdopterin guanine dinucleotide (*bis*-MGD) cofactor is the most abundant one, being present in most bacterial molybdoenzymes.

The first step of Moco biosynthesis, the conversion from 5′GTP to cPMP, is catalyzed by MOCS1A and MOCS1B in mitochondria in eukaryotes, or MoaA and MoaC in bacteria [32] (Table 1). During the conversion of 5′-GTP to cPMP, a (8*S*)-3′,8-cyclo-7,8-dihydroguanosine 5′-triphosphate (3′,8-cH_2_GTP) intermediate is formed. All further steps for the formation of Moco from cPMP are localized in the cytosol in eukaryotes [8,33]. For the formation of MPT from cPMP, two sulfur atoms are incorporated to the C1′ and C2′ positions of cPMP, a reaction catalyzed by MPT synthase. The MPT synthase is a heterotetrameric enzyme consisting of two MOCS2B and two MOCS2A subunits in humans (MoaE and MoaD in *E. coli*). In its active form, MOCS2A, a protein with a ubiquitin-like fold, contains a C-terminal thiocarboxylate group that acts as a direct sulfur donor for the synthesis of the dithiolene group of MPT. This dithiolene group, once formed, serves as a backbone for the ligation of the molybdenum atom in Moco. 

**Figure 3:**
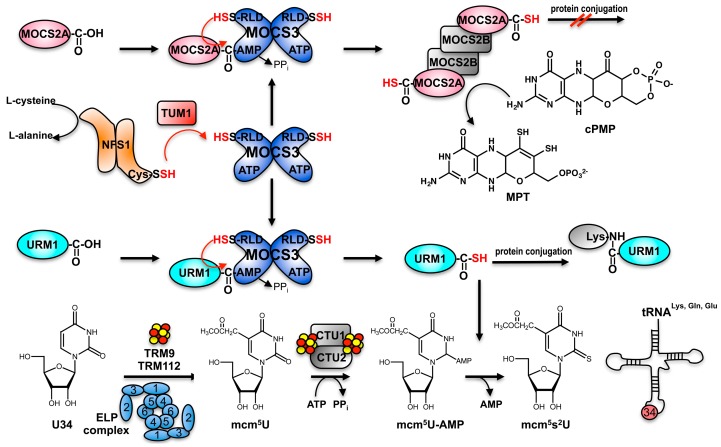
MOCS3 connects the sulfur transfer pathways for the formation of Moco and thiolated nucleosides in tRNA. Formation of the thiocarboxylate group on MOCS2A (upper part). cPMP is converted to MPT by the transfer of two sulfur groups from MOCS2A. MOCS2A is regenerated by MOCS3. The sulfur donor for the persulfide group on MOCS3-RLD is NFS1. Formation of cytosolic 5-methoxycarbonylmethyl-2-thiouridine (mcm^5^s^2^U^34^) in humans (lower part). Proteins of the elongator complex (ELP) and the tRNA methyltransferases TRM9 and TRM112 proteins are involved in the formation of the 5-methoxycarbonylmethyl group (mcm^5^) at the C5 position of U34. MOCS3 activates URM1, which receives the sulfur from NFS1. tRNA is activated by CTU1 and CTU2. URM1 can alternatively be conjugated to target proteins via a lysine–isopeptide bond. RLD: rhodanese-like domain.

For the MPT synthase to act catalytically, it is necessary to regenerate its transferable sulfur in an ATP-dependent reaction, catalyzed by the MOCS3 protein in humans (MoeB in *E. coli*). It was shown that the N-terminal domain of MOCS3 activates the C-terminus of MOCS2A by formation of an acyl-adenylate (Figure 3). *E. coli* MoeB and human MOCS3 are different in a way that MOCS3 contains an additional C-terminal domain at its C-terminus, which is fused to the N-terminal MoeB-like domain and shares amino acid sequence homologies to rhodaneses [34,35]. While in humans, this rhodanese-like domain on MOCS3 acts as a direct sulfur donor for the formation of the thiocarboxylate group on MOCS2A; in *E. coli*, the l-cysteine desulfurase IscS and the 2-thiouridine synthesis protein A (TusA) protein in its persulfide-bound form were identified to act as sulfur-donating enzymes for the generation of a C-terminal thiocarboxylate on MoaD. However, it is also believed that the sulfur for MOCS3 originates from l-cysteine, which is mobilized by NFS1 in the cytosol [36,37]. Thus, NFS1 would mobilize the sulfur for two pathways in humans: Moco biosynthesis in the cytosol and FeS cluster assembly in mitochondria. Crucially, MOCS3 is not only involved in Moco biosynthesis: besides interacting with MOCS2A, MOCS3 also interacts with URM1, which acts as a sulfur acceptor protein involved in the thiolation of some tRNAs [38] (Figure 3). Thus, Moco biosynthesis and tRNA thiolation are directly connected in humans by sharing the sulfur delivery pathway composed of NFS1 and MOCS3. 

In bacteria, the sulfur delivery pathway for Moco biosynthesis is slightly different from the pathway in humans. Here, the sulfur carrier protein TusA directly provides the sulfur for the formation of the thiocarboxylate on MoaD. Since TusA was shown to be additionally the sulfur delivery protein for (c)mnm^5^s^2^U34 formation in tRNAs of *E. coli*, both pathways are directly linked by the TusA protein (described in more detail below). TusA and the rhodanese-like domain of MOCS3, however, do not share any amino acid sequence homologies.

## 3. Sharing Protein Components for Molybdenum Cofactor Biosynthesis and tRNA Thiolation in Eukaryotes: The Thiolation of tRNA in the Cytosol in Eukaryotes

For the thiolation and formation of the mcm^5^s^2^U in the cytosol of eukaryotes, the biosynthesis of the 5-methoxycarbonylmethyl group of the uracil ring (mcm^5^U) is required for efficient 2-thiouridine formation in the cytoplasm [9]. In humans, it was shown that the proteins MOCS3, URM1 (ubiquitin-related modifier), CTU1 and CTU2 are essential for s^2^U34 formation, while proteins of the elongator complex (ELP) synthesize the mcm^5^-group [39]. The ELP pathway consists of the ELP-complex being composed of the six proteins (ELP1–ELP6) in addition to the tRNA methyltransferases TRM9 and TRM112 [40] (Figure 3). The URM1 protein was shown to have a ubiquitin-like β-grasp-fold and contains a conserved C-terminal double glycine-motif on which a thiocarboxylate group is formed for direct sulfur-transfer to mcm^5^U34 in tRNA [20,41,42,43]. The sulfur is transferred to URM1 via MOCS3, which was originally recognized for its role in activation of the MOCS2A protein [38]. For the formation of the thiocarboxylate (-COSH) group at the C-terminal glycine of URM1, the MOCS3-bound persulfide is directly transferred to URM1 [43]. MOCS3 thereby receives its sulfur from the cytosolic form of the l-cysteine desulfurase NFS1 [36]. In detail, MOCS3 activates URM1 in the presence of ATP by formation of activated URM1–AMP (Figure 3). URM1 is further transferred to the persulfide group on Cys412 of MOCS3– rhodanese-like domain (RLD), forming a disulfide bond. The disulfide bond can be cleaved by C239 of MOCS3, which releases thiocarboxylated URM1. URM1–thiocarboxylate further transfers the sulfur onto U34 of the tRNA, aided by the CTU1 and CTU2 proteins under ATP consumption [38]. CTU1 has the PP-motif, several CXXC motifs and zinc-finger motifs putatively for ATPase activity, Fe-S and tRNA binding, respectively [25]. The PP-loop binds ATP that is consumed to activate the target uridine by adenylation. Another conserved cysteine residue in CTU1 is believed to play a key role in sulfur transfer from the URM1–COSH to tRNA [25].

Further, URM1 has been shown to be additionally conjugated to target proteins via a lysine–isopeptide bond, revealing that it is a dual-function protein [39]. Among the targets for urmylation are the components of the tRNA thiolation machinery itself (CTU1, MOCS3) and a deubiquitinating enzyme (USP15), as well as proteins involved in nuclear transport such as the cellular apoptosis susceptibility protein (CAS), which promotes the shuttling of proteins between the cytosol and the nucleus [39]. Here, the role of the protein urmylation is not completely clear to date and might only occur under certain conditions in the cell such as oxidative stress.

## 4. Sulfur Transfer to MOCS3 Involves the NFS1 Protein in the Cytosol

**Figure 4:**
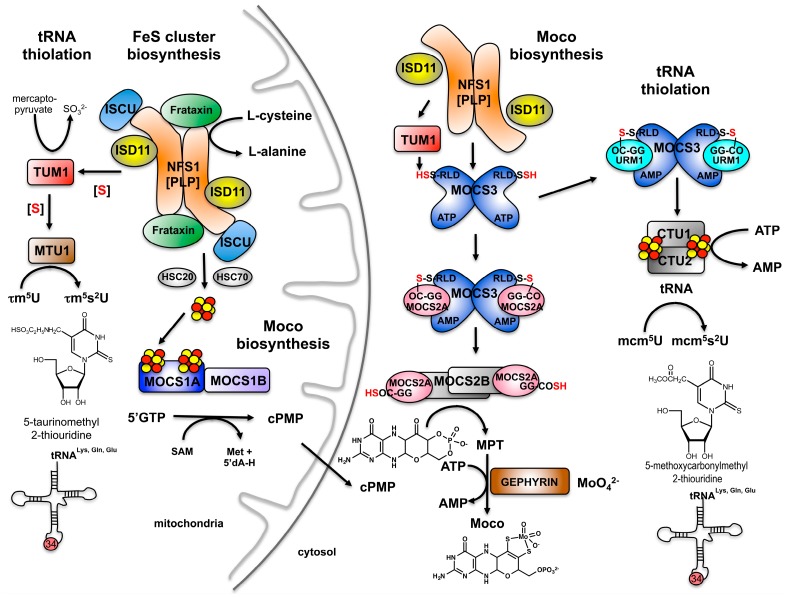
Localization of Moco biosynthesis, FeS cluster biosynthesis and tRNA thiolation in humans. Shown is a scheme of the biosynthetic pathway for Moco biosynthesis, FeS cluster biosynthesis and tRNA thiolation in humans. For Moco biosynthesis, the conversion of 5′GTP to cPMP catalyzed by MOCS1A and MOCS1B, is localized in the mitochondria. This is also the main compartment for FeS cluster biosynthesis in eukaryotes. Synthesized cPMP is further transferred to the cytosol, where all further modification steps are catalyzed. These steps involve the conversion of cPMP to MPT by MOCS2A/MOCS2B and the sulfur transfer by MOCS3, the insertion of molybdate by gephyrin. The main compartment for FeS cluster biosynthesis is the mitochondrion, where NFS1 forms a complex with ISD11, ISCU and Frataxin. Formed FeS clusters are inserted into acceptor proteins such as MOCS1A. In mitochondria, NFS1 also transfers the sulfur to TUM1, a protein involved in the τm^5^s^2^U formation of mitochondrial tRNAs. For TUM1, a dual localization has been revealed. For cytosolic tRNA modification, MOCS3 transfers the sulfur to URM1, which is involved in the formation of mcm^5^s^2^U modified nucleosides in tRNA.

The formation of mcm^5^s^2^U in the cytosol of eukaryotes is directly linked to the two other sulfur-containing cofactors present in the cytosol, Moco and FeS clusters (Figure 4). It was suggested that CTU1 is a FeS cluster-containing protein [25], ensuring tRNA thiolation only when functional FeS clusters are formed [10]. In addition, the MOCS3 protein is a dual-function protein, which is directly shared in tRNA thiolation and Moco biosynthesis serving as an adenylyltransferase not only for URM1, but also for MOCS2A [38]. For Moco biosynthesis, cPMP is transferred from mitochondria to the cytosol, where two sulfur molecules are inserted to build the dithiolene group of MPT [44] (Figure 4). In this reaction, the MPT synthase composed of MOCS2A and MOCS2B is directly involved [35]. MOCS2A thereby acts as direct sulfur donor and inserts two sulfur atoms from two MOCS2A molecules into cPMP, with the sulfur being provided by the C-terminal thiocarboxylate group of MOCS2A. MOCS3 and NFS1 are required for regeneration of the thiocarboxylate group at the C-terminal glycine of MOCS2A in MPT synthase in humans. The sulfur-relay system for Moco biosynthesis for the formation of the dithiolene moiety in MPT was shown to consist of the NFS1-C239 persulfide, the MOCS3-C412 persulfide and the MOCS2A-G88 thiocarboxylate [34,35,36,37]. It has been reported that NFS1 is additionally localized in small amounts in the cytosol, where it interacts with MOCS3 [36] (Figure 4). Thus, cytosolic NFS1 is an important sulfur supplier for both Moco biosynthesis and tRNA thiolation [12]. The interaction and colocalization of NFS1 and MOCS3 was revealed by studies in HeLa cells using Förster resonance energy transfer (FRET), a split-enhanced green fluorescent protein (EGFP) system, immunodetection of fractionated cells and localization studies using confocal fluorescence microscopy [36]. However, while the role of NFS1 in the cytosol for sulfur transfer now seems to be established, open questions still remain. Eukaryotic NFS1 was described to require the protein ISD11 as an essential stabilizing factor for mitochondrial NFS1 activity and FeS cluster biogenesis (refer to below). While the role of NFS1 in the cytosol is required for sulfur transfer to MOCS3, an involvement of ISD11 in this reaction still remains unclear. So far, ISD11 has been described as an essential stabilizing factor for mitochondrial NFS1 activity in eukaryotes [45]. In the absence of ISD11, NFS1 aggregates and conclusively establishes that FeS clusters are not formed in mitochondria [37,46]. Localization studies showed that ISD11 is mainly located in mitochondria and the nucleus in human cells [45]. Thus, the role of ISD11 and its involvement in the interaction of NFS1 and MOCS3 in the cytosol still needs to be revealed. MOCS3 might replace the role of ISD11 as a stabilizing protein to NFS1 in the cytosol. This could imply that cytosolic NFS1 is only involved in Moco biosynthesis and tRNA thiolation in conjunction with MOCS3, while in the absence of ISD11, it has no role in FeS cluster biosynthesis.

In summary, a crosstalk between Moco biosynthesis and cytosolic tRNA thiolation was revealed by the MOCS3 protein, which activates both MOCS2A and URM1. URM1 was shown to have several targets for protein conjugation including MOCS3 and CTU1. In contrast, the role of MOCS2A seems to be restricted to Moco biosynthesis with MOCS2A being unable to conjugate proteins. However, both proteins might have an additional role, since a targeting to the nucleus has been identified. Here, their roles still remain elusive.

## 5. The Route of tRNA Thiolation in Mitochondria

In eukaryotic mitochondria, NFS1 and the mitochondrial tRNA-specific MTU1 are responsible for 2-thiolation of cmnm^5^s^2^U in yeast and 5-taurinomethyl-2-thiouridine (τm^5^s^2^U) in mammals [13] (Figure 4). Here, the taurine modification (τm^5^s^2^U) seems to be restricted to the mitochondria of mammals. MTU1 was shown to be a homologue of the bacterial MnmA protein (described below) [10]. While the 5-taurinomethyluridine (τm^5^U34) is found in mitochondrial tRNAs for Leu and Trp, its 2-thiouridine derivative (τm^5^s^2^U34) is present in mitochondrial tRNAs for Glu, Gln and Lys. These modifications allow tRNAs to precisely recognize their cognate codons and to ensure accurate translation in the mitochondria. Two enzymes, the mitochondrial tRNA translation optimization 1 (MTO1) and the GTP binding protein 3 (GTPBP3) were shown to be responsible for the taurine modification/insertion for the formation of τm^5^U34 [13]. However, the proteins involved in the formation of the taurine group itself remain to be identified. MTU1 catalyzes the subsequent step of the 2-thiolation of τm^5^U34 to form τm^5^s^2^U34. The sulfur for this modification is derived from mitochondrial NFS1 [11].

It was shown that a lack of the τm^5^s^2^U modification in mitochondrial tRNA^Lys^ from individuals with myoclonus epilepsy associated with ragged-red fibers (MERRF) resulted in a marked defect in mitochondrial translation [11]. The lack of the yeast homolog of MTU1 resulted in impaired mitochondrial translation activity and a severe respiratory defect. Moreover, acute knockdown of *MTU1* in HeLa cells reduced the oxygen consumption rate and resulted in a defective mitochondrial membrane potential. Intriguingly, *MTU1* has been implicated in the pathogenesis of reversible infantile liver failure (RILF), a life-threatening condition characterized by acute liver dysfunction during the first 2–4 months after birth. Recent studies on MTU1 knockout mice showed that MTU1 deficiency abolished 2-thiouridine formation in the three tRNAs. Loss of the 2-thiouridine modification resulted in a marked impairment of mitochondrial translation and abnormal mitochondrial structure, showing the importance of this tRNA modification for mitochondrial translation of proteins [47].

## 6. The Dual Role of TUM1 in Mitochondria and the Cytosol?

So far, the sulfur transfer pathway for thionucleosides in tRNA has been mostly investigated in *Saccharomyces cerevisiae*. The sulfurtransferase protein Tum1p (also designated as 3-mercaptopyruvate sulfurtransferase (MPST) or Yor251c) catalyzes the conversion of 3-mercaptopyruvate to pyruvate and a protein-bound persulfide. The yeast protein has been implicated in yeast cytosolic mcm^5^s^2^U34 modification together with Uba4p (MOCS3 homologue) and Urm1p as well as Nfs1p [9]. Furthermore, a conjugation of Urm1p to the antioxidant protein Ahp1p was reported, however, no further protein targets have been identified in yeast so far. Interestingly, Tum1p was shown to be required but not essential for cytosolic tRNA thiolation in yeast [9,48].

Accordingly, the TUM1 protein was suggested to be involved in tRNA thiolation in humans [49]. Interestingly, so far, two uncharacterized TUM1 splice variants have been recently identified, which were designated as TUM1-Iso1 and TUM1-Iso2. The two splice variants revealed a different cellular localization, with TUM1-Iso1 being exclusively localized in the cytosol, while TUM1-Iso2 showed a dual localization both in the cytosol and in mitochondria. The respective interaction partners of the two isoforms proved to be more different. While TUM1-Iso1 interacted with both NFS1 and MOCS3 in the cytosol, TUM1-Iso2, in contrast, exclusively interacted with MOCS3 in the cytosol but not with NFS1. In mitochondria, TUM1-Iso2 showed the expected interaction with NFS1. Conclusively, this implied distinct roles for each TUM1 isoform in the sulfur transfer processes in the cell. Still, numerous questions remain on the function of TUM1. Splice variants for Tum1p were not reported in yeast so far and tRNA thiolation differs between yeast and humans. Human mitochondrial tRNAs contain a taurine modification, which is not present in yeast mitochondrial tRNA, containing the cmnm^5^U34 modification instead. Furthermore, in yeast, Moco biosynthesis is not present, making yeast an ideal and simple system to study tRNA thiolation as a separate pathway. Therefore, major differences between tRNA thiolation and Moco biosynthesis exist from human to yeast. In humans, the protein network appears more complex since Moco biosynthesis is an essential cellular pathway, which is required for the activity of sulfite oxidase (SUOX) as a detoxifying enzyme. 

## 7. Sharing Sulfur Mobilization Routes for Molybdenum Cofactor Biosynthesis and tRNA Thiolation in Prokaryotes: The Pathway for (c)mnm^5^s^2^U Modification of tRNA in Bacteria

**Figure 5:**
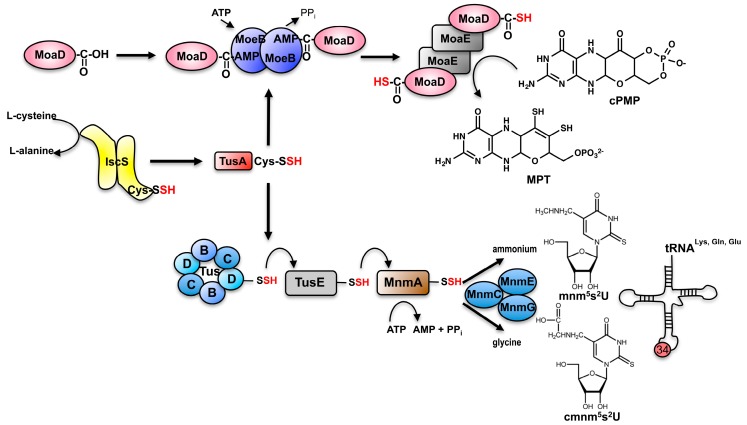
IscS and TusA connect the sulfur transfer pathway for the synthesis of Moco and for tRNA thiolation in *E. coli*. Formation of the thiocarboxylate group on MoaD (upper part). cPMP is converted to MPT by the transfer of two sulfur groups from the C-terminal thiocarboxylate of the MoaD. Regeneration of the MoaD thiocarboxylate group by MoeB. MoaD receives its sulfur by a persulfide-sulfur relay system consisting of IscS and TusA. Formation of cmnm^5^s^2^U and mnm^5^s^2^U in *E. coli* (lower part). Sulfur relay system from IscS via TusA, TusD (in the TusBCD complex), TusE to MnmA, which binds and activates the tRNA by adenylation. After formation, s^2^U is further modified to form cmnm^5^s^2^U or mnm^5^s^2^U by MnmG, MnmE and MnmC.

While the thiolation of tRNA in the cytosol of human cells is linked to the biosynthesis of Moco by sharing the same sulfur transfer mechanism using NFS1 and MOCS3, the τm^5^s^2^U modification of nucleosides of tRNA in mitochondria of humans rather resembles the (c)mnm^5^s^2^U tRNA thiolation pathways identified in bacteria. For the formation of (c)mnm^5^s^2^U in tRNA for Lys, Gln, Glu in *E. coli*, a sulfur-relay system was identified including the initial sulfur mobilization by the l-cysteine desulfurase IscS and the proteins TusA, TusBCD, TusE and MnmA [50] (Table 1 and Figure 5). So far, the five *E. coli* genes *tusA*, *tusB*, *tusC*, *tusD* and *tusE*, were identified to be essential for the formation of s^2^U34 in tRNAs. Among them, *tusB*, *tusC* and *tusD* are encoded on a single operon and their gene products form the TusBCD complex composed as a dimer of a heterotrimer (αβγ)_2_. Efficient 2-thiouridine formation can be achieved in vitro using purified tRNA incubated with purified proteins IscS, TusA, the TusBCD complex, TusE and MnmA [50]. TusA thereby directly interacts with IscS, stimulates its desulfurase activity and directs the sulfur flow to 2-thiouridine formation. After the formation of a persulfide on Cys19 of TusA, TusA transfers the sulfur onto Cys278 of TusD in the TusBCD complex. TusE then interacts with TusBCD and accepts the sulfur from TusD. TusE subsequently transfers the sulfur to Cys199 of MnmA. MnmA is a member of the ATP-pyrophosphatase family, which bears a PP-loop as a signature motif [51]. MnmA binds tRNA and ATP and activates the bound tRNA by forming an activated acyl-adenylated intermediate on U34. Subsequently, a nucleophilic attack by the persulfide sulfur of Cys199 of MnmA generates a tRNA thiocarbonyl group and releases AMP [51,52,53]. Finally, the thiolated tRNA dissociates from MnmA after a nucleophilic attack by Cys102 on Cys199. The formed disulfide bridge within MnmA needs to be reduced before the next catalytic cycle can occur. For the formation of the (c)mnm group, the MnmG–MnmE complex and MnmC are essential. The mnm^5^ modification and the s^2^U modification in bacterial tRNAs were shown to occur independently. Depending on the substrate of MnmEG, two different routes of modification at the C5 position of uridine are possible. Either uridine is converted into mnm^5^U (5-methylaminomethyluridine) using ammonium as a substrate or cmnm^5^U (5-carboxymethylaminomethyluridine) using glycine as substrate [54]. In turn, MnmC can convert cmnm^5^ to nm^5^U using FAD as a cofactor, which is bound to its C-terminal domain. The N-terminal part of MnmC is capable of donating a methyl group to nm^5^U forming the mature mnm^5^U modification in a SAM (*S*-adenosyl-methionine) dependent fashion. 

In comparison, for the 5-taurinomethyluridine (τm^5^U) modification in human mitochondria, the MnmE and MnmG homologues were identified to be the GTPBP3 and MTO1 proteins, respectively, which incorporate taurine instead of the 5-methylamino group into tRNA [55]. In the subsequent thiolation step, the human MTU1 protein is involved, which was shown to be the homolog of *E. coli* MnmA. Thus, the (c)mnm^5^s^2^U34 modification in bacteria resembles the route for τm^5^s^2^U34 modification in mitochondria of mammals rather than the mcm^5^s^2^U34 modification in the cytosol of eukaryotes.

## 8. TusA Links Moco Biosynthesis to tRNA Thiolation in Bacteria

The function of TusA as sulfur transferase is shared between the route of tRNA thiolation and Moco biosynthesis in bacteria such as *E. coli*. In Moco biosynthesis, the dithiolene group of MPT is formed after insertion of two sulfur atoms to the C1′ and C2′ atoms of cPMP (Figure 5). The direct sulfur donor in this reaction is the thiocarboxylate group of MoaD–COSH, present in the MPT synthase complex. The formation of the thiocarboxylate group on MoaD directly requires MoeB [56]. The MoaD modification reaction resembles the first step of the activation of ubiquitin for the ubiquitin-dependent protein degradation system in eukaryotes [57]. MoaD thereby has a ubiquitin fold, and MoeB shares amino acid sequence identities with ubiquitin-activating enzyme E1. In the course of the reaction, MoeB and MoaD form the tetrameric (MoaD–MoeB)_2_ complex in which an acyl-adenylate group is formed at the C-terminal glycine of MoaD under ATP consumption [56,58]. In its activated form, sulfur is directly transferred to MoaD–AMP in the (MoaD–MoeB)_2_ complex. MoaD–COSH then reassociates with MoaE_2_ to form the active MPT synthase heterotetramer [59,60]. 

It was shown that in *E. coli*, l-cysteine serves as the origin of the MPT dithiolene sulfurs in a reaction involving the l-cysteine desulfurase IscS in addition to the TusA protein [61,62]. TusA was originally identified to have a role in tRNA thiolation (described above). It has been suggested that TusA transfers the sulfur from IscS directly to MoaD in a sulfur relay step. Conclusively, this would imply that TusA–SSH interacts with two sulfur acceptor proteins, namely MoaD for MPT formation and TusD for (c)mnm^5^s^2^U34 thiolation. 

So far, detailed studies showed that a deletion of *tusA* causes a pleiotropic effect on several cellular pathways in *E. coli*, not only including tRNA thiolation and Moco biosynthesis, but also the enhanced susceptibility of viral infection inhibition by programmed ribosomal frameshifting. These pleiotropic effects of a deletion in *tusA* were suggested to be caused by changes in the FeS cluster concentration in the cell, thereby revealing the link of FeS cluster availability on tRNA thiolation and for Moco biosynthesis [62]. Studies showed that elevated levels of TusA in *E. coli* decreased the level of FeS clusters. Consequently, when FeS clusters are limited, FeS containing proteins such as MoaA are inactive which directly results in a decreased activity of molybdoenzymes. Surprisingly, on the other side, overexpression of IscU (scaffold for FeS cluster assembly) also reduced the level of active molybdoenzymes in *E. coli*. This observation was explained to be based on the elevated complex formation of IscU with IscS, thereby limiting IscS availability for interaction with other proteins such as TusA. Conclusively, it has been suggested that TusA has a contribution in regulating the availability of IscS for the different sulfur transfer pathways. This implies that the absence of TusA increases the availability of IscS for FeS cluster assembly [62]. As a consequence, sulfur transfer from IscS to other biosynthetic pathways such as Moco or thiolated tRNA are reduced. Conclusively, this emphasizes that the sulfur transfer pathways to sulfur containing biomolecules such as Moco biosynthesis, FeS cluster assembly and thiolation of tRNAs are tightly connected and likely regulated at a cellular level by the availability of their acceptor proteins. 

## 9. The Thiolation of tRNA in Thermophiles

It has been revealed that in thermomphilic bacteria, the thermostability of tRNA is effected by post-transcriptional modifications. These tRNA modifications include a 5-methyl-2-thiouridine (m^5^s^2^U) thiomodification at position 54 in the T-loop of tRNA. Intriguingly, its biosynthesis pathway resembles that of the cytosolic mcm^5^s^2^U34 modification in eukaryotes. The 2-thio-modification of m^5^s^2^U54 was shown to play an important role in translation under growth conditions at elevated temperatures. The studies were performed in thermophilic organisms such as *Thermus thermophilus* or *Pyrococcus furiosus* in addition to *Aquifex aeolicus*, where almost all tRNA species were found to be modified forming m^5^U54 or m^5^s^2^U54 [63,64,65]. By increasing the cultivation temperature in these organisms, it was shown that the ratio of the m^5^U54 or m^5^s^2^U54 modification increased to the benefit of the m^5^s^2^U54 thiolation [64,66]. Therefore, the thio-modification has been suggested to be essential for the survival of these thermophilic bacteria at high temperatures. 

The pathway for the m^5^s^2^U54 modification includes the enzymes for 2-thioribothymidine synthesis TtuA, TtuB and TtuC. TtuB thereby is a small ubiquitin-like sulfur carrier protein which is activated by TtuC [67]. Similar to the eukaryotic CTU1/MOCS3/URM1 pathway described above, the C-terminal glycine of TtuB is modified by TtuC under ATP consumption to form TtuB-COAMP. In the subsequent sulfur transfer step, the thiocarboxylate group on TtuB is formed. TtuB–COSH then transfers the sulfur atom to the position 54 in the T-loop of tRNA [10]. In this step, the TtuA protein is involved, which is a PP-loop containing protein. It was suggested that TtuA activates the target uridine 54 in tRNA by forming an acyl-adenylated U54 nucleoside intermediate under ATP consumption [68]. The PP-loop is a common motif of ATP pyrophosphatases, including the tRNA modification enzymes MnmA in bacteria and CTU1 in eukaryotes [69]. However, the tRNA modification pathway of thermophiles rather resembles the CTU1/URM1/MOCS3 modification system of eukaryotes, and not the MnmA modification system of bacteria. The combining sulfur transfer mechanism used for tRNA thiolation is therefore that of a thiocarboxylate group formed on a small ubiquitin-like carrier protein. Further, TtuB can be covalently attached to acceptor proteins such as TtuA, suggesting that thiouridine synthesis is regulated by TtuB protein-conjugation, thus, representing the first ubiquitin-like conjugation system in bacteria [70].

## 10. Archaeal Proteins Involved in tRNA Thiolation

The biosynthesis of s^2^U34 in cytosolic tRNA in eukaryotes is based on a sulfur transfer mechanism with a protein-bound thiocarboxylate intermediate. This pathway is similar to a sulfur transfer pathway that was recently identified in archaea [71]. Genetic analysis in *Haloferax volcanii* identified that the small archaeal modifier protein (SAMP) proteins are ubiquitin-like proteins with roles in tRNA thiolation and Moco biosynthesis. SAMP1 was identified to be a MoaD-like protein that acts together with UbaA (MoeB-homologue in *H. volcanii*). 

SAMP2, in contrast, was identified to be involved in tRNA thiolation, and an interaction with UbaA for thiouridine formation has been identified [72]. Further, NcsA (CTU1-homologue of *H. volcanii*) is also required for 2-thiouridine formation. The SAMP proteins were additionally identified to be covalently conjugated to proteins. SAMP2 was shown to be covalently conjugated to target proteins including UbaA, NcsA, and Tum1 [73], implying that the tRNA thiolation machinery itself is regulated by SAMP2 modification. In conclusion, the studies identified the SAMP proteins as being the origin of ubiquitin-like protein modifiers in archaea [73].

## 11. A Short Introduction to FeS Cluster Assembly as Essential Cofactors for Both tRNA Thiolation and Moco Biosynthesis

**Figure 6:**
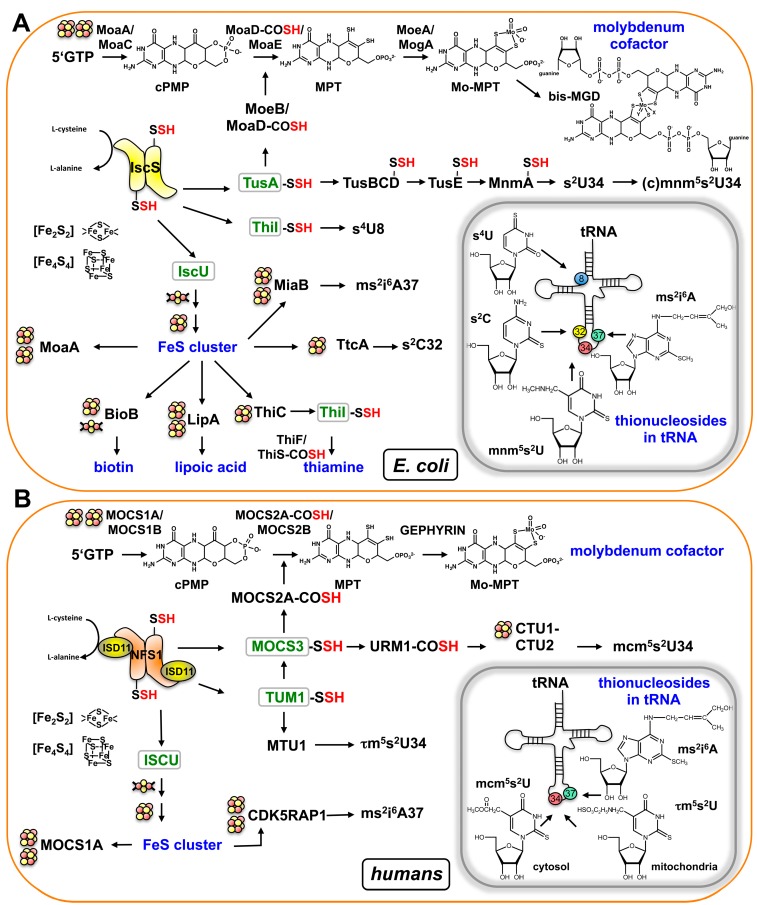
Sulfur transfer routes for the biosynthesis of sulfur-containing molecules in *E. coli* (**A**) and humans (**B**). Detailed descriptions are given in the text. Acceptor proteins that receive the sulfur from the l-cysteine desulfurase are marked in green. Proteins that contain FeS clusters are marked by one or two cluster symbols. The locations of the modified nucleosides in tRNA are shown in the inset boxes. –SSH: persulfide, –COSH: thiocarboxylate.

From the descriptions above, the link of both the thiolation of tRNA and Moco biosynthesis to FeS cluster biosynthesis was revealed. A direct link is provided by the l-cysteine desulfurase, a protein which provides the sulfur for multiple sulfur-containing cofactors such as Moco, lipoic acid, biotin, thiamine as well as for the addition of thionucleosides s^2^C32 and ms^2^i^6^A37 in tRNA (Figure 6). Accordingly, IscS in prokaryotes was shown to interact with a number of proteins for sulfur delivery in different pathways, namely (i) IscU in complex with the protein CyaY, ferredoxin (Fdx) and IscX for FeS cluster formation; (ii) TusA for either the (c)mnm^5^s^2^U34 modification of tRNA or the biosynthesis of the Moco, in addition to (iii) ThiI for either the synthesis of thiamine or the s^4^U8 modification of tRNA [3] (Figure 6A). Different binding sites for some of these molecules were mapped on *E. coli* IscS.

In *E. coli*, IscS is encoded by a gene that is part of a larger operon, *iscRSUA-hscBA-fdx-iscX*, containing all essential components for the assembly of FeS clusters [74]. In the initial step, IscS interacts with IscU which serves as a scaffold protein for FeS cluster assembly [75,76]. Both proteins form a S-shaped heterotetrameric complex, thereby making IscU accessible to receive the persulfide sulfur from IscS. The iron source for nascent FeS cluster formation has not been identified yet, however, several proteins have been discussed as potential candidates [77]. Initially, one [Fe_2_S_2_] cluster is formed per IscU monomer, inducing a conformational change within the IscU protein that leads to a decreased stability of the IscS–IscU interaction [3]. During FeS cluster assembly, electrons are required for persulfide reduction allowing FeS formation and/or reductive coupling of two [Fe_2_S_2_] clusters to form one single [Fe_4_S_4_] cluster on IscU. These electrons are most likely provided by Fdx [78,79,80]. Two heat shock cognate chaperones, HscA and HscB, catalyze cluster release in an ATP-dependent reaction to carrier proteins (such as A-type carriers as IscA) or the final apo-target proteins [81]. Further, recent investigations have shown that the small acidic proteins CyaY and IscX assist in FeS cluster formation in vivo [77]. They are able to bind IscS, however, their specific role in this process remains controversial [76].

In eukaryotes, the mitochondria constitute the main compartment for the biosynthesis of FeS clusters. In humans, the main proteins required for FeS cluster biosynthesis are NFS1, ISD11, ISCU and Frataxin (FXN), which form the quaternary core complex [82,83,84]. ISD11 thereby is exclusively present in eukaryotes and was described as a stabilizing factor for NFS1, being essential for the activity in FeS cluster formation in mitochondria. After the synthesis of the FeS cluster on ISCU, the cluster is transferred by the help of carrier proteins to mitochondrial acceptor proteins [85]. It has been proposed for yeast and mammals, that the mitochondrial ABC transporter Atm1 (ATP cinding cassette subfamily B member 7 [ABCB7] in mammals) exports either fully formed FeS clusters or a special form of sulfur that is required for cytosolic FeS cluster biosynthesis [86,87]. Deletions of Atm1 in yeast *S. cerevisiae* or patient mutations, have been proposed for the export of a “sulfur compound” from mitochondria, which contributes to cytosolic FeS cluster assembly (CIA) via proteins known as Tah18 (NDOR1 in mammals) and Dre2 (CIAPIN1 in mammals) [88]. After FeS cluster formation in the cytosol, these are transferred to cytosolic and nuclear acceptor proteins, with the help of NUBP1, NUBP2, NARFL and CIAO1 proteins of the CIA pathway [89]. 

Importantly, FeS cluster-containing proteins are involved in Moco biosynthesis and the thiolation of tRNA. In bacteria, it was shown that the synthesis of the thiogroup of tRNAs containing s^2^C32 or ms^2^i^6^A37 are dependent on FeS cluster-containing proteins (Figure 6A). In *E. coli*, these tRNA thiolations involve TtcA for the synthesis of s^2^C and MiaB for ms^2^i^6^A modification. TtcA contains only one [Fe_4_S_4_] cluster [90], while MiaB contains two [Fe_4_S_4_] clusters and is a member of the radical SAM superfamily of proteins [91]. In contrast, the synthesis of s^4^U8 and (c)mnm^5^s^2^U34 is FeS cluster independent in bacteria. In eukaryotes, the cytosolic CTU1 protein involved in modification is a FeS cluster-containing protein, making cytosolic mcm^5^s^2^U34 formation a FeS-dependent pathway (Figure 6B). Further, in Moco biosynthesis, the MOCS1A protein in eukaryotes or the MoaA protein in bacteria belongs to the group of radical SAM proteins, which require a [Fe_4_S_4_] cluster for their catalytic activity. In the case of MOCS1A, the protein binds an additional [Fe_4_S_4_] cluster at its C-terminus, being involved in binding of the substrate 5′-GTP.

## 12. Conclusions

It has been shown that the sulfur insertion into biomolecules is a complex pathway involving multiple protein components in the cell (Figure 6A,B). Interestingly, sulfur transfer for distinct and unrelated biomolecules such as Moco and thiolated nucleosides in tRNA share the same protein components, which are conserved among bacteria, eukaryotes and archaea. On the one hand, sulfur is transferred in the form of a persulfide from a donor protein to an acceptor protein, usually including the formation of an activated acyl-adenylated intermediate. On the other hand, a persulfide can also be transferred from a donor forming a thiocarboxylate on the acceptor protein, and the thiocarboxylate sulfur is either inserted into the biomolecule directly or into an activated acyl-adenylated intermediate. One mechanism for the sulfur transfer for xm^5^s^2^U tRNA thiolation exists in bacteria, eukaryotes and archaea, which involves a thiocarboxylate on a ubiquitin-like modifier. A second thiocarboxylate/ubiquitin-like modifier independent mechanism is present solely in bacteria and in the mitochondria of eukaryotes, which uses the persulfide chemistry. Further, it has to be pointed out that the two different pathways for xm^5^s^2^U formation are either separated into two compartments (mitochondria and cytosol) as in eukaryotes or do not simultaneously exist in one bacterial or archaeal species. Whether there is a reason for this separation of the pathways in different species needs to be investigated in future studies.

In total, the thiolation of tRNA molecules is a complex chemical process involving multiple sulfur carrier proteins. Extensive studies on s^2^U34 formation and Moco biosynthesis revealed that shared mechanisms of sulfur-relay systems exist, which use the same protein components. “Sharing” of the same protein components implies that a regulated system has to exist that mediates the sulfur flow into the designated biomolecule as an acceptor. 

Most likely, the cell tries to reduce the presence of an excess of free sulfide in the cell to avoid unspecific interference with cellular processes. Further, multiple protein components involved in each transfer pathway bear significant advantages for the cell. Firstly, cells can fine-tune the direction of the sulfur flow by adjusting the available concentration of the proteins. Secondly, sulfur can be stored in a non-toxic and assessable way, which might facilitate the adaptation to changing growth conditions or internal factors caused e.g., by oxidative stress. These open questions need to be investigated in future studies.

## Figures and Tables

**Table 1:** Overview of the reactions catalyzed by proteins for molybdenum cofactor (Moco) biosynthesis and transfer (tRNA) thiolation in bacteria, humans and yeast.

*Escherichia coli*	Humans	Yeast	Reaction Catalyzed
**Persulfide sulfur providing enzyme**			
IscS	NFS1	Nfs1	l-cysteine desulfurase
-	ISD11	Isd11	stabilizing protein for NFS1
**Moco biosynthesis**			
MoaA	MOCS1A	-	formation of (3′,8-cH_2_GTP) from 5′-GTP, binds two [Fe_4_S_4_]
MoaC	MOCS1B	-	formation of cPMP from (3′,8-cH_2_GTP)
MoaD	MOCS2A	-	sulfur transfer as –COSH to cPMP, formation of MPT
MoaE	MOCS2B	-	binding of cPMP, formation of MPT
MoeB	MOCS3	-	adenylation of MPT synthase small subunit
-	MOCS3-RLD	-	rhodanese-like domain for formation of MOCS2A–COSH
TusA	-	-	sulfur transferase for formation of MoaD–COSH
MogA	GEPHYRIN-G	-	formation of MPT–AMP
MoeA	GEPHYRIN-E	-	molybdate insertion into MPT–AMP, Mo–MPT formation
**xm^5^s^2^U34**			
MnmA	MTU1	Mtu1	thiouridylase for the formation of (c)mnm^5^s^2^U (*E. coli*), or tm^5^s^2^U in human mitochondria or cmnm^5^s^2^U in mitochondria in yeast, adenylation of tRNA
TusA	-	-	sulfur relay system involved in the formation of (c)mnm^5^s^2^Umodified nucleosides in *E. coli*
TusBCD			
TusE			
-	TUM1	Yor251c	3-MPST for formation of τm^5^s^2^U34 in human mitochondria or cmnm^5^s^2^U34 in mitochondria in yeast
-	MOCS3	Uba4	mcm^5^s^2^U34 modified nucleosides in the human and yeast cytosol, adenylation and URM1–COSH formation
-	URM1	Urm1	thiocarboxylate sulfur transfer for mcm^5^s^2^U34 in the cytosol of humans and yeast, conjugated to proteins
-	CTU1-CTU2	Ncs6-Ncs2	thiouridylase for mcm^5^s^2^U34 formation in the cytosol of humans and yeast, adenylation of tRNA, CTU1/Ncs6 is a [Fe_4_S_4_] cluster-containing protein
**s^4^U8**			
ThiI	-	-	involved in the formation of s^4^U8 and thiamin in *E. coli*
**s^2^C32**			
TtcA	-	-	thiotransferase for s^2^C32 in *E. coli*, binds one [Fe_4_S_4_]
**ms^2^i^6^A37**			
MiaB	CDK5rap1	-	methylthiotransferase for the formation of ms^2^i^6^A37 in *E. coli* or in mitochondria in humans, binds two [Fe_4_S_4_]

IscS: Iron sulfur cluster synthesis protein S; NFS1: Nitrogen fixation 1 homolog; ISD11: Iron sulfur biogenesis desulfurase interacting protein 11; MoA–E: Molybdenum A–E; MOCS1–3: Molybdenum cofactor synthesis protein 1–3; cPMP: Cyclic pyranopterin monophosphate; MPT: Molybdopterin; RLD: Rhodanese-like domain; TusA–E: 2-thiouridine synthesis protein A–E; MnmA: 5-methylaminomethyl-2-thiouridine synthase A; TUM1: tRNA thiouridin modfication protein 1; MPST: Mercaptopyruvate sulfurtransferase; Uba4: Ubiquitin-like protein activator 4; CTU1–2: Cytoplasmic tRNA 2-thiolation protein 1–2; Nsc: Needs Cla4 to survive; ThiI: Thiamin synthesis protein I; TtcA: tRNA(cytosine32)-2-thiocytidine synthetase A; MiaB: tRNA (N6-isopentenyl adenosine(37)-C2)-methylthiotransferase B; CDK5rap1: Cyclin-dependent-like kinase 5 repressor/activator site-binding protein 1; Urm1: Ubiquitin-related modifier 1; MTU1: Mitochondrial tRNA-specific 2-thiouridylase; Yor251c: systematic name for TUM1 in yeast; GTP: guanosine triphosphate.
